# Targeted temperature management in cardiac arrest: survival evaluated by propensity score matching

**DOI:** 10.1186/s13049-017-0373-1

**Published:** 2017-03-16

**Authors:** Eirik A. Buanes, Karl O. Hufthammer, Jørund Langørgen, Anne-Berit Guttormsen, Jon-Kenneth Heltne

**Affiliations:** 1 Department of Anaesthesia and Intensive Care, Haukeland University Hospital, P.O. Box 1400, NO-5021 Bergen, Norway; 2 Department of Clinical Medicine, University of Bergen, Bergen, Norway; 3 Centre for Clinical Research, Haukeland University Hospital, Bergen, Norway; 4 Department of Heart Diseases, Haukeland University Hospital, Bergen, Norway

**Keywords:** Cardiac arrest, Outcome, Targeted temperature management, Propensity score matching, Intensive care

## Abstract

**Background:**

Targeted temperature management in cardiac arrest was introduced following evidence of increased survival from two controlled trials published in 2002. We wanted to investigate whether the introduction of targeted temperature management to clinical practice had increased the survival of cardiac arrest patients at Haukeland University Hospital, Norway.

**Methods:**

We included 336 unresponsive patients admitted to the emergency department between December 2003 and December 2008 with return of spontaneous circulation following out-of-hospital cardiac arrest in the analysis. A propensity score model was developed to evaluate the survival of patients receiving intensive care treatment including targeted temperature management, compared with intensive care treatment not including targeted temperature management.

**Results:**

Estimation of the treatment effect revealed an increase of 57 days (95% CI: 12–103, *p* = 0.01) in restricted mean survival during the first year after cardiac arrest for intensive care treatment including targeted temperature management.

**Discussion:**

As with all observational studies, bias is probable. However, propensity score methodology has been used in order to reduce bias and establish causality. Although residual confounding is likely, our interpretation is that TTM increased survival for comatose OHCA patients in our hospital because survival increased well beyond the level of significance.

**Conclusion:**

The introduction of targeted temperature management to clinical practice is likely to have increased survival for unresponsive patients following out-of-hospital cardiac arrest.

**Electronic supplementary material:**

The online version of this article (doi:10.1186/s13049-017-0373-1) contains supplementary material, which is available to authorized users.

## Background

Targeted temperature management (TTM) at 32–34 degrees Celsius (°C) following cardiac arrest (CA) was introduced to clinical practice based on two controlled studies published in 2002, one randomised and one pseudorandomised [1, 2]. Both studies found that TTM improved survival and neurologic function following CA compared with standard care. In 2011, a meta-analysis questioned the robustness of these findings, and, 2 years later, a randomised controlled trial of TTM at 33 °C compared to TTM at 36 °C was published [3, 4]. No difference in survival was found between the two groups. A subgroup analysis published in 2015 found no difference as regards the neurologic outcome in the same two groups [5].

The cause of the discrepancy between these studies is not known. It could be that TTM has no effect in clinical practice, where the strict criteria of a randomised controlled study do not apply. Another view is that improved intensive care treatment over the past decade has removed the conditions for which TTM had an effect. Perhaps TTM was of greater importance when the attention to cerebral perfusion pressures, body temperature and blood gas values was less stringent.

In order to shed light on this development, we decided to analyse registry data from the introduction of TTM at Haukeland University Hospital. This would allow us to estimate the effects of TTM in clinical practice at a time when intensive care treatment was similar to that in the initial studies. Patients treated with TTM were compared with those not treated with TTM. We applied propensity score matching in order to generate comparable groups and establish causality. The primary objective was to test the hypothesis that TTM improves survival after CA. The secondary objective was to investigate the effect of TTM on cognitive function in CA survivors.

## Methods

### Trial design

The study is a retrospective observational study from two intensive care units at Haukeland University Hospital, Norway.

### Patients

**Fig. 1:**
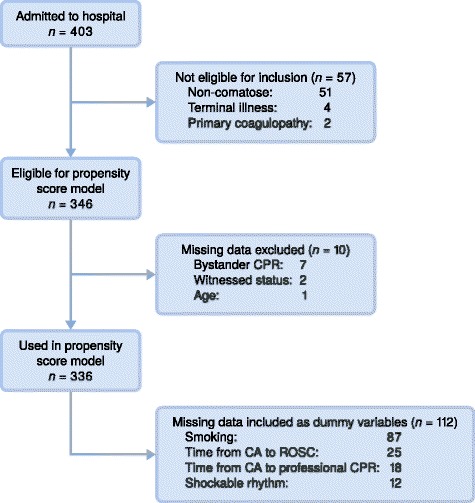
Flowchart presenting included and excluded patients

Epidemiology of CA in the Bergen area has previously been described [6]. In December 2003, local guidelines were introduced for the pre-hospital and in-hospital treatment of CA survivors. New features in the guidelines included the avoidance of fever, a more structured approach to intensive care treatment and the introduction of TTM. The guidelines specified that TTM was indicated in comatose CA survivors between 18 and 80 years old where professional cardiopulmonary resuscitation (CPR) was commenced within 15 min of CA and the return of spontaneous circulation (ROSC) was achieved within 60 min of CA. The guidelines further specified that patients with non-cardiac cause of CA, terminal illness, severe comorbidity, in need of nursing care on a daily basis or with coagulopathy should not be offered TTM. The guidelines were not strictly enforced, and the ultimate decision regarding TTM or no TTM was taken by individual clinicians. This is one of the prerequisites for propensity score matching, which allowed us to retain patients with these characteristics in both TTM and non-TTM groups. The patients considered for inclusion in this study were survivors of out-of-hospital cardiac arrest (OHCA) who were available for TTM in the emergency department of Haukeland University Hospital between December 2003 and December 2008. Patients with a Glasgow Coma Score (GCS) > 8 and patients with coagulopathy or terminal illness were excluded (Fig. 1).

### Intervention

TTM was induced using axillary and femoral ice bags before hospital admission, and cold saline after admission, until invasive TTM was established. The CoolGard Temperature Management System with ICY Catheters (Zoll Medical Corporation, MA, USA) was used for invasive TTM. Patients were cooled to 33 °C at a rate of 0.5 °C per hour and maintained at 33 °C for 24 h before rewarming at a rate of 0.5 °C per hour to 37 °C. Seven patients included in the intervention group received surface cooling because invasive cooling was unavailable. In these seven patients, TTM was established using active cooling garments in five patients, ice bags and cold towels in one patient and ice bags alone in one patient.

### Outcome

The primary outcome was restricted mean survival time, defined as the number of days alive from CA until death limited to a maximum of 365 days after CA. The secondary outcome was cerebral function measured by the cerebral performance category (CPC) scale upon hospital discharge, a scale from 1 to 5, where 1 is good recovery and 5 is death [7]. CPC was scored retrospectively by registrars based on information in the medical records. No criteria existed regarding the withdrawal of life-sustaining therapy.

### Statistics

Patients were identified and data was collected retrospectively from local quality databases and medical records between 2009 and 2011. Data was stored in a restricted access database. Initial comparisons between treatment groups were performed using *t*-tests for continuous variables and chi-squared test for discrete variables. All baseline variables affecting both treatment and outcome are potential confounders and were considered for inclusion in the propensity score model. Due to the small dataset, variables only weakly associated with outcome were excluded [8]. Potential confounders included were age, gender, shockable primary rhythm, witnessed CA, bystander CPR, time from CA to professional CPR, time from CA to ROSC, smoking status, known diabetes mellitus, known hypertension and previous myocardial infarction. Because of low counts in some categories, smoking was recoded from four to three categories and hypertension from three to two categories. Time from CA to ROSC was winsorised to 60 min (i.e. longer times were replaced by 60 min).

For predictors with less than 10 missing values, the observations with missing data were discarded. For continuous predictors with more than 10 missing values, missing values were replaced by fixed values and indicator variables for ‘missing’ were included in the model. For categorical predictors with more than 10 missing values, ‘missing’ was treated as a separate category [9, 10]. See Fig. 1 for a flowchart of the data used.

**Fig. 2:**
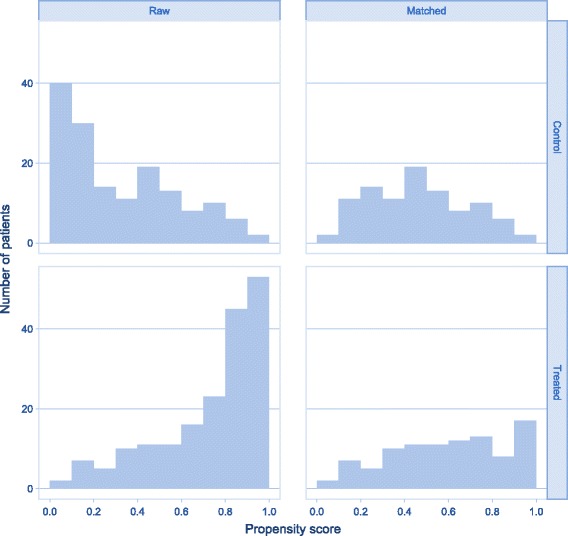
Distribution of propensity scores between TTM-treated patients and controls

The propensity score was modelled using logistic regression. To ensure that the model was flexible enough to accurately predict treatment, we used second-degree polynomial terms for all three continuous variables. The distribution of the propensity score in the treatment groups was examined to ensure adequate overlap (Fig. 2). Balance checks were based on examining means, proportions and distributions of predictors before and after matching (Table 3) [8].

Non-treated subjects were matched 1:1 to treated subjects within callipers, using a best-first (‘greedy’) algorithm. The calliper width for matching was set to 10% of the standard deviation of the logit of the propensity score since the common rule of 20% did not adequately balance the predictors.

Survival was compared using mean values restricted to a maximum of 365 days [11]. The treatment effect was estimated using a linear mixed-effects model with a random intercept for each matching pair. For comparison purposes, we also report the results from a naïve *t*-test (ignoring any confounders), and from an ordinary least squares model, where we adjust for confounders by including the same predictors in the same form as in the propensity score model.

Stata SE version 14.0 (StataCorp LP, Texas, USA) and R version 3.2.3 with the ‘nonrandom’ package version 1.42 were used for data analysis [12, 13].

## Results

A total of 403 comatose CA patients were available for TTM in the study period. Exclusions due to study criteria numbered 57, while exclusions due to missing data numbered 10 (Fig. 1). This left 336 patients eligible for inclusion in the propensity score model, 183 (54%) of them TTM-treated cases and 153 non-TTM controls. A crude comparison between treated cases and controls revealed significant differences in age, gender, primary rhythm, presumed cause of arrest, resuscitation and medical history (Tables 1 and 2). The propensity score model developed to balance these differences satisfied the overlap assumption, i.e. treated cases and controls with similar propensity scores were available for matching. We successfully matched 96 treated cases with controls. The propensity scores had similar distributions in cases and controls after matching, and most baseline covariates were sufficiently matched (Fig. 2, Table 2, Additional file 1: Table S5). This indicates that the propensity score model was adequately specified and suited for causal inference. The estimation of treatment effects revealed that survival in the first year after CA (restricted mean comparison) increased by 57 days (95% CI: 12–103, *p* = 0.01) in TTM-treated cases. The mean CPC value at discharge was reduced by 0.5 (95% CI: 0.1–1.0, *p* = 0.02) in TTM-treated cases (Table 3).

**Table 1:** Demographic and medical characteristics among study patients before matching

	Control (*n* = 153)		Treated (*n* = 183)		
	Count/[Mean]	Percent^a^/(SD)	Count/[Mean]	Percent^a^/(SD)	*P*-value
Mean age [years] with (95% CI)	[64]	(20)	[59]	(16)	0.01
Male gender	86/153	56%	140/183	77%	<0.001
Primary shockable rhythm	41/143	29%	132/181	73%	<0.001
Medical history					
Previous myocardial infarction	42/153	27%	52/183	28%	0.94
Heart failure	26/116	22%	25/179	14%	0.086
Hypertension	48/126	38%	62/178	35%	0.64
Lung disease	37/119	31%	32/181	18%	0.01
Diabetes	25/122	20%	25/180	14%	0.17
Kidney disease	13/118	11%	4/179	2%	<0.001
Malignancy	16/135	12%	9/178	5%	0.047
Smoke	65/97	67%	113/152	74%	0.27
Hypercholesterolemia	19/67	28%	33/154	21%	0.35
Presumed cause of arrest					
Acute myocardial infarction	43/153	28%	104/183	57%	<0.001
Previous myocardial infarction	7/153	5%	24/183	13%	0.01
Other heart disease	6/153	4%	10/183	5%	0.69

^a^The percentage values are based on patients with non-missing data

**Table 2:** Distribution of potential confounders before and after matching

	Before matching					After matching				
	Control (*n* = 153)		Treated (*n* = 183)			Control (*n* = 96)		Treated (*n* = 96)		
	Count/	Percent^a^/	Count/	Percent^a^/		Count/	Percent^a^/	Count/	Percent^a^/	
	[mean]	(SD)	[mean]	(SD)	*P*-value	[mean]	(SD)	[mean]	(SD)	*P*-value
Age	[64]	(20)	[59]	(16)	0.01	[63]	(18)	[61]	(17)	0.63
Male Gender	86	56%	140	77%	<0.001	59	61%	70	73%	0.12
Shockable rhythm					<0.001					0.09
No	102	67%	49	27%		56	58%	41	43%	
Yes	41	27%	132	72%		38	40%	53	55%	
*(Missing)*	10	7%	2	1%		2	2%	2	2%	
Witnessed CA	111	73%	164	90%	<0.001	76	79%	80	83%	0.58
Bystander CPR	72	47%	130	71%	<0.001	52	54%	56	58%	0.66
Time CA to professional CPR	[14.6]	(25.5)	[11.2]	(16.8)	0.15	[7.1]	(5.6)	[8.0]	(5.5)	0.32
*(Missing)*	12	8%	6	3%	0.11	6	6%	6	6%	1.00
Time CA to ROSC	[27.2]	(16.5)	[26.7]	(16.1)	0.77	[24.4]	(13.8)	[22.7]	(12.6)	0.38
*(Missing)*	9	6%	16	9%	0.43	6	6%	8	8%	0.78
Smoking					0.001					0.44
No	32	21%	39	21%		23	24%	18	19%	
Ex-smoker	28	18%	47	26%		19	20%	19	20%	
Smoker	37	24%	66	36%		27	28%	37	39%	
*(Missing)*	56	37%	31	17%		27	28%	22	23%	
Diabetes Mellitus					<0.001					0.69
No	97	63%	155	85%		73	76%	79	82%	
Type 1	5	3%	5	3%		4	4%	2	2%	
Type 2	20	13%	20	11%		16	17%	12	12%	
*(Missing)*	31	20%	3	2%		3	3%	3	3%	
Hypertension					<0.001					0.83
No	78	51%	116	63%		56	58%	57	59%	
Yes	48	31%	62	34%		33	34%	34	35%	
*(Missing)*	27	18%	5	3%		7	7%	5	5%	
Previous myocardial infarction	42	27%	52	28%	0.94	30	31%	29	30%	1.00

*SD* standard deviation, *CA* cardiac arrest, *CPR* cardiopulmonary resuscitation, *ROSC* return of spontaneous circulation

^a^The percentage values are based on patients with non-missing data

**Table 3:** Estimated restricted mean survival after cardiac arrest, and estimated mean CPC score after discharge

	TTM treated cases		Non-TTM controls		Difference				*P*-value
	*n*	Mean	*n*	Mean	Mean	95% CI			
Survival time (days)									
Crude	183	199	153	69	131	97	to	164	<0.001
Propensity-score matched	96	142	96	84	57	12	to	103	0.01
Regression-adjusted	183	–	153	–	52	14	to	91	0.008
CPC score									
Crude	183	3.0	151	4.4	−1.3	−1.7	to	−1.0	<0.001
Propensity-score matched^a^	95	3.7	95	4.2	−0.5	−1.0	to	−0.1	0.02
Regression-adjusted	183	–	151	–	−0.5	−0.9	to	−0.1	0.01

^a^CPC data was missing for one observation in one of the matched pairs. The pair was excluded from the analysis

## Discussion

The propensity score analysis shows that intensive care treatment with TTM compared to intensive care treatment without TTM significantly increased the restricted mean survival by 57 days (95% CI: 12–103) during the first year after CA.

The important question is whether the increase in survival is due to TTM or due to bias. The data in this study were collected at a time when new technology was introduced. As with all observational studies designed this way, bias is probable. Patients with a better prognosis may have been selected for TTM more frequently than patients with a worse prognosis, and withdrawal of care may have been more aggressive in patients not selected for TTM. An important aspect of this study is the introduction of propensity score methodology in order to reduce bias and establish causality. While not often used in CA research, the method is common in epidemiologic research. Moreover, the method may prove valuable in the analysis of the large quantity of data accumulating in various CA registries.

Propensity score methodology reduces bias by using two important mechanisms. First, the overlap assumption must be fulfilled. This means that there must be a sufficient number of patients with the same probability of treatment in both the treatment and the control group. The propensity score assigned to each individual in the sample is the estimated probability of treatment given the baseline covariates. The second mechanism is balance checks of the baseline covariates. Propensity score methodology states that all variables which are measured before allocation of treatment and which affect both treatment and outcome, i.e. all variables which are possible confounders, must be included in the propensity score model. The balance analysis checks that these variables are distributed equally between TTM and non-TTM groups [8]. We have included a comprehensive list of possible confounders. Although the balance in our matched sample is not perfect, any differences are small, and this greatly reduces any bias the confounders might introduce to the estimation of treatment effect (Table 2). Therefore, we believe that the majority of the estimated treatment effect is due to TTM rather than confounding.

Restricted mean survival time was chosen as the primary outcome instead of the commonly used hazard ratio. The hazard ratio measure relies on the assumption of proportional hazards, i.e. it assumes a constant hazard ratio over time. The advantage of the restricted mean survival time is that it measures survival over the entire period from inclusion to follow-up, and does not depend on the proportional hazards assumption [11].

We decided prior to the analysis that follow-up of 1 year would be reasonable in CA. Survival beyond this point is probably more dependent on factors unrelated to CA than on the effects of CA. Treatment by TTM increased survival by an estimated 57 days during the first year after CA. This is a significant difference in a robust outcome measure. An impression of the magnitude of the difference can be gained from a study comparing the restricted mean survival time to hazard ratios in well-known cancer studies [14]. The hazard ratios in the RE01 trial in advanced kidney cancer, the GOG111 trial in advanced ovarian cancer and the IPASS trial in lung cancer were 0.73/0.75/0.73, respectively. Restricted mean survival times in the same three studies were 0.9 months/3 months/9 months, respectively. This indicates that the increase in restricted mean survival time of 57 days in our study is clinically significant.

The effect of TTM on cognition has been debated [15]. Our analysis shows a mean reduction in the CPC score of 0.5 points (95% CI: 0.1–1.0) for TTM-treated cases upon hospital discharge. When interpreting this result, it should be borne in mind that death is scored as five on the CPC. Since TTM-treated cases have better survival than non-TTM controls, it is likely that part of the reduction in the CPC score is due to improved survival. Further studies are needed to ascertain the effect of TTM on brain function in CA survivors [16].

A total of 15 control patients had axillary and femoral ice bags placed prior to admission, and one control patient received infusions with cold saline after admission (Table 4). This may have influenced the estimation of treatment effects. However, a recent study of patients with out-of-hospital CA found no effect on survival or neurologic recovery of pre-hospital induction of TTM using cold saline versus standard induction after admission [17].

**Table 4:** Distribution of treatment characteristics among study patients before matching

	Control (*n* = 153)		Treated (*n* = 183)		
	Count	Percent	Count	Percent	*P*-value
Ice bags prior to admission	15	10%	176	96%	<0.001
Cold saline after admission	1	1%	168	92%	<0.001
Intensive care treatment					
Sedative drugs	71/135	53%	172/172	100%	<0.001
Muscle relaxant drugs	6/153	4%	133/183	73%	<0.001
Antiepileptic drugs	5/153	3%	51/183	28%	<0.001
Dialysis	0/153	0%	4/183	2%	0.18

**Fig. 3:**
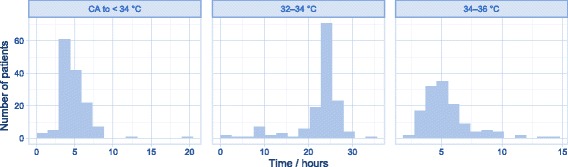
Distribution of time in various temperature zones in TTM-treated patients

Stringent criteria for inclusion and exclusion in our study, combined with clearly defined outcome measures and robust statistical methods, are the main strengths of the study. However, this cannot outweigh the limitations of a retrospective observational design [18]. Selection bias may be hidden among unmeasured baseline covariates not included in our data. We also had to use a narrow calliper in order to ensure comparable groups. This reduces the number of cases available for matching and thus reduces the generalisability of the results. The matching did not completely balance the covariates (Table 2), indicating some probability of residual confounding. However, any bias in the estimated treatment effect should be greatly reduced compared to a crude analysis. Since new treatment was introduced in a non-blinded fashion, it is likely that TTM patients were assigned better trained and more dedicated personnel. It is also probable that TTM patients were given extra attention in other aspects of their treatment, spurred by the introduction of new technology. Data on body temperature are limited, and restricted to the treatment group (Fig. 3). As a result, no per-protocol analysis was possible, and nor do we know to what extent fever was present. The neurological outcome (CPC) was scored in a non-blinded fashion. In essence, residual confounding is likely, and we must assume that some of the estimated treatment effect is due to other factors than the assigned treatment.

However, survival increased well beyond the level of significance in a relatively small sample. Our interpretation of the result is that TTM in fact did increase survival for comatose OHCA patients in our hospital between December 2003 and December 2008. Interestingly, our hospital was also part of the recent randomised TTM study, which found no difference between TTM at 33 and 36 °C in patients enrolled between November 2010 and January 2013 [4]. An explanation for this might be that TTM is of importance only when brain resuscitation is marginal, i.e. when cerebral perfusion pressures are low and blood glucose and blood gas values are abnormal. A review of intensive care treatment from historical TTM patients compared with present TTM patients could uncover if this is the case.

## Conclusions

The study shows that intensive care treatment including TTM increased survival for unresponsive CA patients compared to intensive care treatment without TTM in our hospital.

## Additional file

Additional file 1: Table S5. Distribution of the presumed cause of cardiac arrest before and after matching. (XLSX 10 kb)

## Abbreviations

CA: Cardiac arrest
CI: Confidence interval
CPC: Cerebral performance category
CPR: Cardiopulmonary resuscitation
GCS: Glasgow coma score
OHCA: Out-of-hospital cardiac arrest
ROSC: Return of spontaneous circulation
TTM: Targeted temperature management
